# CD73 is a hypoxia-responsive gene and promotes the Warburg effect of human gastric cancer cells dependent on its enzyme activity

**DOI:** 10.7150/jca.62387

**Published:** 2021-09-03

**Authors:** Xiaopeng Cao, Ziman Zhu, Yi Cao, Jia Hu, Min Min

**Affiliations:** 1 Department of Gastroenterology, the First Medical Center of Chinese PLA General Hospital, Beijing, China; 2 Department of Hepato-Pancreato-biliary Surgery, the First Medical Center of Chinese PLA General Hospital, Beijing, China; 3 Department of Global Health, Milken Institute School of Public Health, the George Washington University, Washington DC, USA; 4 Department of Oncology, the Fifth Medical Center of Chinese PLA General Hospital, Beijing, China

**Keywords:** CD73, Warburg effect, Gastric cancer, NT5E, Hypoxia

## Abstract

**Background:** The Warburg effect is closely associated with malignant phenotypes and poor prognosis in gastric cancer. CD73 is a glycosylphosphatidylinositol (GPI) anchored cell surface protein that functions as an oncogene in a variety of human cancers. However, the relationship between CD73 and the Warburg effect has yet to be fully understood.

**Methods:** Integrative analysis was performed to identify glycolysis-related genes in gastric cancer. Loss-of-function and gain-of-function are performed to demonstrate the roles of CD73 in gastric cancer cell proliferation and glycolysis. Cell biological, molecular, and biochemical approaches are used to uncover the underlying mechanism.

**Results:** In this study, we find that CD73 is a glycolysis-associated gene and is induced by hypoxia in gastric cancer. Genetic silencing of CD73 reduces gastric cancer cell proliferation and glycolytic ability. Opposite effects were observed by CD73 overexpression. Importantly, pharmacological inhibition of CD73 activity by APCP inhibits tumor growth, which can be largely compromised by the addition of adenosine, suggesting an enzyme activity-dependent effect of CD73 in gastric cancer. Furthermore, hijacking tumor glycolysis by 2-DG or galactose largely abrogated the oncogenic roles of CD73, indicating that CD73 promotes tumor growth in a glycolysis-dependent manner in gastric cancer. By the subcutaneous xenograft model, we confirmed the promotive roles of CD73 in regulating cell proliferation and glycolysis in gastric cancer.

**Conclusions:** This study provides strong evidence of the involvement of CD73 in the Warburg effect and indicates that it could be a novel antitumor strategy to target tumor metabolism in gastric cancer.

## Introduction

Gastric cancer is one of the most common malignant tumors in the world. The incidence and mortality of gastric cancer in East Asia and China remain high 1, 2. Due to the low frequency of regular gastroscopy, insidious early symptoms, poor results of radiotherapy and chemotherapy, the mortality rate of gastric cancer has remained high, the clinical outcome of patients with advanced and metastatic gastric cancer is still not optimistic 3. Therefore, in-depth studying of the molecular mechanism involved in gastric cancer occurrence and development and developing effective diagnosis and treatment methods are urgently needed.

Due to the hypoxia and low pH microenvironment, the glucose metabolism of tumor cells is different from that of normal cells, which is manifested by high glycolysis but not oxidative phosphorylation and known as the Warburg effect 4, 5. Abnormal energy metabolism especially the Warburg effect has emerged as a key feature of most tumor cells 6. Increased Warburg effect is associated with many oncogenic phenotypes in cancers, such as rapid cell proliferation, drug resistance, and stemness 7. Therapies targeting the Warburg effect have a profound inhibitory effect on tumor progression. In gastric cancer, glycolytic proficiency negatively affects survival outcomes of metastatic gastric cancer patients treated with paclitaxel-ramucirumab systemic therapy 8. Notably, several reports have documented the regulators of the Warburg effect in gastric cancer, such as MACC1 9, FBP1 10, WTAP 11, and MUC16 12. However, the molecular mechanisms underlying the Warburg effect in gastric cancer remain unclear.

CD73, also known as NT5E (5'-Nucleotidase Ecto), is glycosylphosphatidylinositol-anchored cell surface protein and has been suggested to be dysregulated in most types of human cancer 13, 14. CD73 catalyzes the hydrolysis of AMP into adenosine and phosphate, and CD73-generated adenosine was verified to be involved in carcinogenesis, cancer apoptosis escape, and therapeutic resistance 15, 16. Moreover, CD73 also functions as a signal and adhesive molecule that can regulate cell interaction with extracellular matrix components, such as laminin and fibronectin, to mediate the invasive and metastatic properties of cancers. In gastric cancer, CD73 was originally found to be methylated in primary gastric cancer tissue. Subsequently, CD73 gene mutation was observed in gastric cancer metastasis 17. Notably, CD73 overexpression in gastric cancer tissues and serums was noticed and associated with a poor clinical outcome in patients with gastric cancer 18. Moreover, CD73 was reported to promote tumor metastasis by modulating RICS/RhoA signaling and EMT in gastric cancer 16. However, the link between CD73 and the Warburg effect remains largely unknown.

In this study, we show that highly expressed CD73 is induced by hypoxia in gastric cancer. Genetic silencing or pharmacological inhibition of CD73 decreases the colony formation ability and glycolytic capacity of gastric cancer cells. Tumor growth advantage induced by CD73 is glycolysis-dependent and enzyme activity-dependent. Taken together, our study suggests that CD73 plays an important role in the Warburg effect by catalyzing the AMP breakdown into adenosine.

## Materials and methods

### Gene expression analysis

For analysis of differentially expressed genes in gastric cancer samples, data from The Cancer Genome Atlas (TCGA) and the Genotype-Tissue Expression (GTEx) were used and analyzed by the GEPIA database 19. TCGA RNA-seq data of gastric cancer samples (n = 408) was used for correlation analysis and the correlation coefficient was determined by the Spearman method.

### Cell culture and reagents

The eight human gastric cancer cell lines: AGS, BGC-823, HGC-27, MKN-45, and SGC-7901 were acquired from ATCC or the Institute of Biochemistry and Cell Biology, Chinese Academy of Science (Shanghai, China). These cancer cells were routinely cultured in RPMI-1640 medium or DMEM, supplemented with 10% (v/v) of fetal bovine serum (FBS, Gibco, Grand Island, NY, USA) and 1% (v/v) streptomycin-penicillin (Sigma-Aldrich, Shanghai, China). All cells were maintained at 37°C in a 5% CO_2_ incubator. APCP (CD73 inhibitor) and adenosine were acquired from Sigma (Shanghai, China). Galactose (S3849) and 2-Deoxy-D-glucose (2-DG, S4701) were obtained from Selleck (Shanghai, China).

### Cell transfection

Short hairpin RNAs (shRNAs) against CD73 gene were transfected along with a three plasmid system (pPACKH1-REV, pPACKH1-GAG, and pVSV-G) into HEK293T cells using Lipofectamine 2000 (Invitrogen, Carlsbad, CA, USA) according to the manufacturer's protocols. The shRNA sequences were shown as follows: shRNA-1, 5′-GATCCGGAATCGTTGGATACACTTCCTTCAAGAGAGGAAGTGTATCCAACGATTCCTTTTTTG-3′; shRNA-2, 5′-GATCCGCCGCTTTAGAGAATGCAACATTCAAGAGATGTTGCATTCTCTAAAGCGGCTTTTTTG-3′. Conditioned medium (CM) containing retroviral particles was collected at 48 h after transfection, and filtered through 0.45-μm filters. After incubating with conditioned medium containing retroviral particles and 5 μg/ml polybrene (Sigma-Aldrich, H9268, St. Louis, MO) for two days, the target cells were selected by 2 μg/ml puromycin for 2 weeks to yield shRNA-expressing cells. For siRNAs-mediated HIF1α knockdown experiment, gastric cancer cancer cells were seeded into 6-well plate at 70-80% HIF1α confluency and were transfected with 15 μmol siRNAs by Lipofectamine 2000 (11668019, Invitrogen, USA) according to the manufacturer's instructions. The sequences of si-HIF1α were shown as follows; si-HIF1α-1, GAGGAAGAACUAAAUCCAA; si-HIF1α-2, UGAUACCAACAGUAACCAA. Scramble non-target siRNAs were used as negative controls. The knockdown efficiency was detected by western blotting. For CD73 overexpression experiment, CD73 coding gene was cloned into the pcDNA3.1 expression vector. Next, the pcDNA3.1-CD73 and control vector plasmids were transfected into gastric cancer cells using Lipofectamine 2000 (11668019, Invitrogen, USA) according to the manufacturer's protocol.

### Real-time quantitative PCR

Total RNA was extracted from gastric cancer cell lines with RNAiso Plus reagent (Takara, Japan) according to the manufacturers' instruction and then reverse transcribed with a PrimeScript RT-PCR kit (Takara, Japan). cDNA was amplified by PCR with 10 μl reaction system using SYBR-Green PCR Master Mix in a Fast Real-time PCR 7500 System (Applied Biosystems, USA). All real-time qPCR reactions were completed in triplicate. The primer sequences used in this study were shown as follows: *GLUT1* forward, 5′-ATTGGCTCCGGTATCGTCAAC-3′, *GLUT1* reverse, 5′-GCTCAGATAGGACATCCAGGGTA-3′; *HK2* forward, 5′-TTGACCAGGAGATTGACATGGG-3′, *HK2* reverse, 5′-CAACCGCATCAGGACCTCA-3′; *ENO1* forward, 5′-GCCGTGAACGAGAAGTCCTG-3′, *ENO1* reverse, 5′-ACGCCTGAAGAGACTCGGT-3′; *PKM2* forward, 5′-AAGGGTGTGAACCTTCCTGG-3′, *PKM2* reverse, 5′-GCTCGACCCCAAACTTCAGA-3′; *LDHA* forward, 5′-ATGGCAACTCTAAAGGATCAGC-3′, *LDHA* reverse, 5′-CCAACCCCAACAACTGTAATCT-3′. *GAPDH* forward, 5′-CTGGGCTACACTGAGCACC-3′, *GAPDH* reverse, 5′-AAGTGGTCGTTGAGGGCAATG-3′. *GAPDH* was served as an internal control.

### Western blotting

Gastric cancer cells with indicated treatment were rinsed with cold PBS before treated with RIPA lysis buffer (50 mM Tris, 0.15 M NaCl, 1 mM EGTA, 1% NP40, 0.25% SDS, pH 7.4) containing protease and phosphatase inhibitors. Cell pellets were incubated for 30 min on ice to homogenize fully and then total proteins in the supernatant of cell lysates were collected by centrifuging at 4°C at 12,000 rpm for 10 min. Total protein concentrations were measured by the BCA protein assay kit (Pierce, USA) before the experiment. Then, standard western blotting assays (SDS-PAGE) were used to analyze protein expression. The antibodies used for western blotting analysis in this study included: anti-CD73 (Abcam, ab133582, 1:1,000 dilution), anti-HIF1α antibodies (Abcam, ab51608, 1:1,000 dilution), and anti-β-actin antibody (Sigma, A2228, 1:5,000 dilution). After incubation with corresponding species-specific secondary HRP-conjugated antibodies, the target protein bands were visualized by an ECL imaging system.

### Glucose uptake and lactate production

Glucose and lactate levels in the culture medium were detected using Glucose Assay kit (Biovision, Milpitas, CA, USA) and Lactate Assay kit (Biovision, Milpitas, CA, USA), respectively. In brief, gastric cancer cells were seeded at 5 x 10^5^ cells in 6-cm cell culture dishes and allowed to grow for 24 h with indicated treatment. After 24 h, the culture medium was collected and centrifuged at 2,000 rpm for 5 min to obtain the supernatant without cell debris. Finally, glucose and lactate assays were performed according to the manufacturer's instructions, and colorimetric density was assessed using a Multifunctional microplate reader.

### Plate colony formation assay

Gastric cancer cells were resuspended and plated in 6-well plates at a density of 500 cells per well and allowed to grow for 10-14 days with indicated treatment. Then the cells were fixed with 4% paraformaldehyde for 15 min at room temperature and stained with 0.5% crystal violet for 15 min. The experiment was performed in triplicate.

### Chromatin immunoprecipitation-PCR

Chromatin Immunoprecipitation (ChIP) experiment was performed in gastric cancer cells under 20% O_2_ and 1% O_2_ conditions. In brief, gastric cancer cells with indicated treatment were fixed with 1% formaldehyde for 10 min at room temperature followed by incubation with 0.125 M glycine for 10 min. Next, cells were collected, washed with ice-cold PBS three times, resuspended in lysis buffer, supplemented with protease inhibitor cocktails, and sonicated to obtain chromatin fragments of about 500-1,500 bp. The supernatants were obtained and incubated with anti-HIF1α antibody (F7425) and Dynabeads Protein G at 4°C overnight. The beads were washed, and the precipitated chromatin complexes were collected, purified, and de-crosslinked. Finally, the precipitated DNA fragments were analyzed by RT-PCR.

### Animal studies

Male BALB/c athymic nude mice (5-6 weeks old and weighing 18-20 g) were purchased from the Jiesijie Animal Center (Shanghai, China). To establish the gastric cancer xenograft model, 2 × 10^6^ stable CD73-knockdown and sh-Ctrl gastric cancer cells were suspended in 100 μl PBS and inoculated subcutaneously into the flanks of nude mice. After 4 weeks, the mice were sacrificed, and the tumors were excised and subjected for further analysis. These experiments were performed in full accordance with the recommendations in the Guide for the Care and Use of Laboratory Animals of the National Institutes of Health, and all the animal experiments were approved by the Animal Experimental Ethics Committee.

### Immunohistochemistry

Standard procedure of Immunohistochemistry (IHC) analysis was performed. In brief, tissue sections (4 μm) were firstly deparaffinized, followed by citrate-based antigen retrieval and blocking with 0.2% endogenous peroxidase. After blocking with 10% bovine serum albumin (BSA, Sangon, Shanghai, China) for 45 min at room temperature, the slides were incubated with primary antibodies at 4 °C overnight. For Ki67 staining, sections from xenograft tumor tissues were incubated with a monoclonal mouse anti-Ki67 antibody (Cell Signaling Technology; 1:8,000 dilution; #2586), followed by staining with an HRP-conjugated secondary antibody (Abcam; 1:300 dilution; ab6728). Immunoreactivity was generated by the DAB system. Finally, sections were counterstained by hematoxylin, and images were analyzed with a NIKON microscope.

### Statistical analysis

Data were represented as mean ± standard deviation (SD). GraphPad 6.0 (GraphPad Software Inc., San Diego, CA) was used for statistical analysis. The correlation of gene expression was evaluated by Spearman's correlation. P-values were calculated by two-tailed unpaired Student's t-test or one-way ANOVA by two-tailed post hoc Dunnett's multiple comparison test. P < 0.05 was considered to be statistically significant.

## Results

### Identification of glycolysis-associated genes in gastric cancer

**Figure 1:**
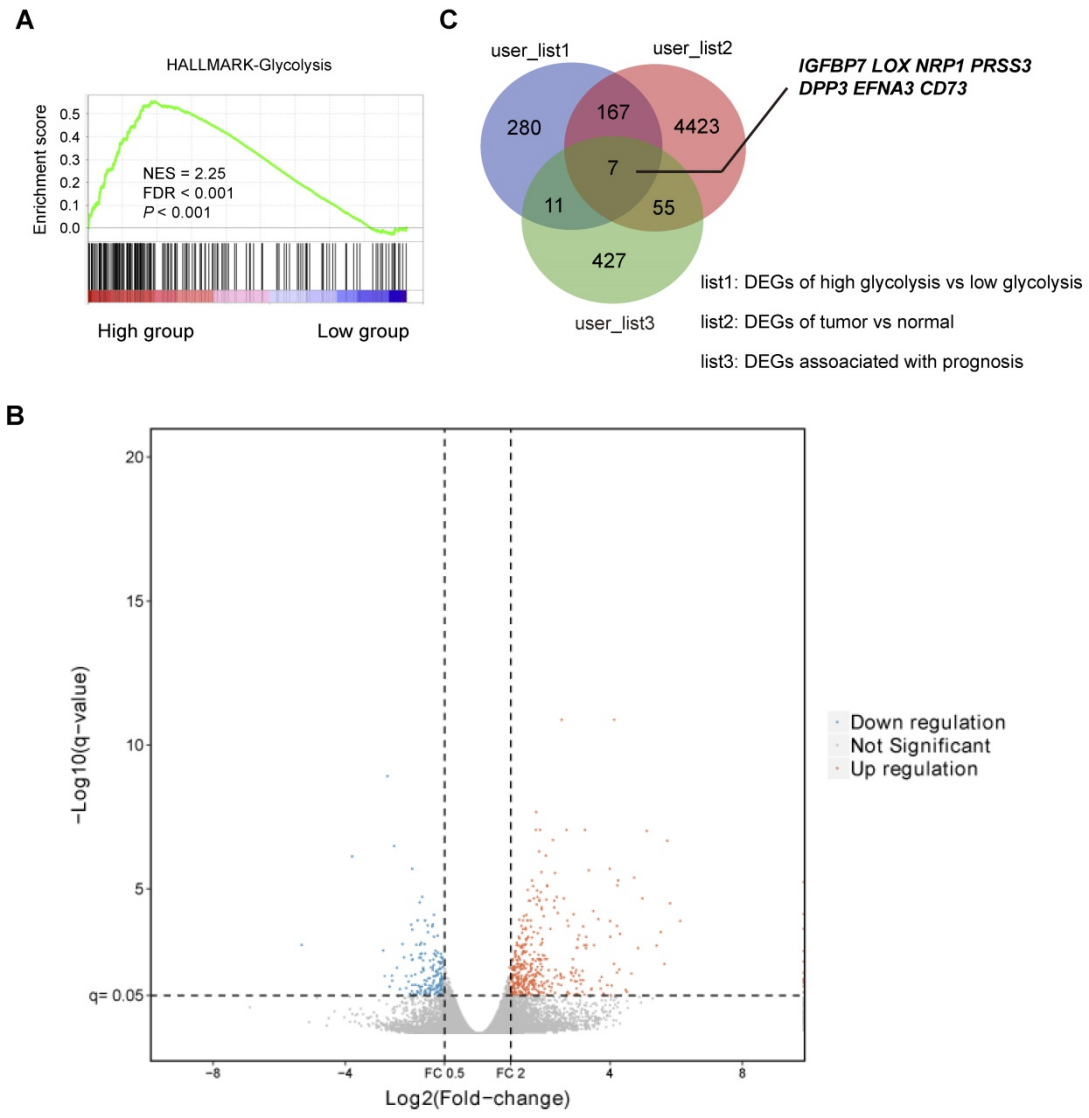
** Identification of glycolysis-associated genes in gastric cancer.** (**A**) Gene set enrichment analysis (GSEA) on Hallmark glycolysis gene sets across the glycolysis-low and glycolysis-high samples. NES, normalized enrichment score (NES); false discovery rate (FDR) was set at 0.25. (**B**) Volcano plot of differentially expressed genes (DEGs) between gastric cancer samples clustered in glycolysis-low and glycolysis-high groups. (**C**) Venn diagram showed DEGs related to glycolysis, DEGs between tumor and normal tissues, and genes associated with a better or a poor prognosis in gastric cancer.

To investigate the differentially expressed genes (DEGs) related to gastric cancer cell glycolysis, the expression profiles of mRNA data of stomach adenocarcinoma (STAD) were acquired from The Cancer Genome Atlas (TCGA) and used for subsequent analysis. Based on a glycolysis gene signature reported previously 20, the STAD cohort was divided into two groups: glycolysis-low (n = 204) and glycolysis-high (n = 204). Gene set enrichment analysis (GSEA) showed that HALLMARK glycolysis gene signature was significantly enriched in the glycolysis-high samples (**Figure 1A**). The heatmap of differentially expressed genes between glycolysis-high and glycolysis-low groups was shown in **Figure 1B**. Among the identified DEGs, we found that 7 genes (*IGFBP7*, *LOX*, *NRP1*, *PRSS3*, *DPP3*, *EFNA3*, and *CD73*) were also differentially expressed in tumor tissues and associated with a poor or better prognosis in patients with gastric cancer (**Figure 1C**). Among these genes, no reports regarding the role of *CD73* in reprogrammed metabolism were found. Therefore, *CD73* was of particular interest to investigate.

### CD73 is induced by hypoxia in gastric cancer

**Figure 2:**
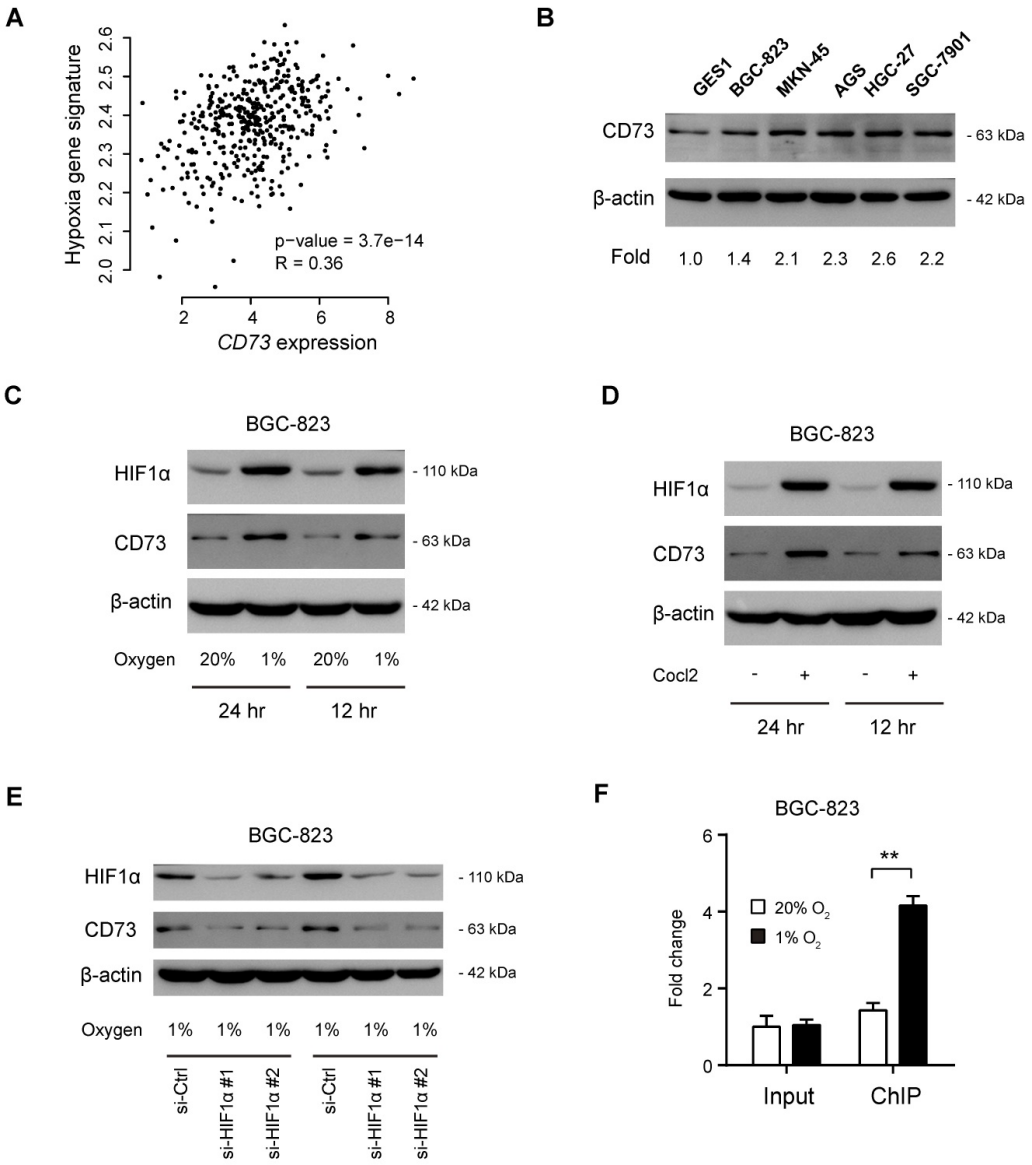
** CD73 is induced by hypoxia in gastric cancer.** (**A**) Correlation analysis of CD73 expression and hypoxia gene signature in gastric cancer; data were obtained from TCGA cohort. (**B**) Western blotting analysis of CD73 expression in gastric cancer cell lines and GES1 cell line. (**C**) BGC-823 cells were cultured under hypoxia (1% O_2_) and normoxia (20% O_2_) for 12 or 24 h, followed by detection of CD73 expression with western blotting. (**D**) BGC-823 cells were cultured with the treatment of 100 μM CoCl_2_ or not for 12 or 24 h, followed by detection of CD73 expression with western blotting. (**E**) Under hypoxic culture condition (1% O_2_), CD73 expression in BGC-823 cells in the presence or absence of HIF1α knockdown was analyzed by western blotting. (**F**) Chromatin immunoprecipitation-PCR validation of the regulatory role of HIF1α in CD73 expression. Input and ChIP cycle threshold (Ct) values were normalized separately to control as 1. **P < 0.01.

By coexpression analysis, we found a close correlation between CD73 and hypoxia gene signatures (**Figure 2A**). By western blotting analysis of CD73 in gastric cancer cell lines, we observed that CD73 was highly expressed in gastric cancer cells compared with the nonmalignant GES1 cells (**Figure 2B**). To determine whether CD73 is induced by hypoxia in gastric cancer, we cultured BGC-823 cells under both normoxic and hypoxic conditions (**Figure 2C**). As a result, CD73 expression was significantly boosted by hypoxia at protein levels (**Figure 2C**), indicating a regulatory role of HIF1α in CD73 expression. Similarly, a chemical inducer of HIF1α, CoCl_2_, phenocopied the effect induced by hypoxia (**Figure 2D**). As the next line of evidence, genetic silencing of HIF1α largely attenuated hypoxia-induced CD73 expression (**Figure 2E**). Moreover, chromatin immunoprecipitation data showed that HIF1α interacted directly with CD73 gene promoters under hypoxia (**Figure 2F**). Therefore, highly expressed CD73 may be induced by hypoxia in gastric cancer.

### CD73 regulates the Warburg effect in gastric cancer

**Figure 3:**
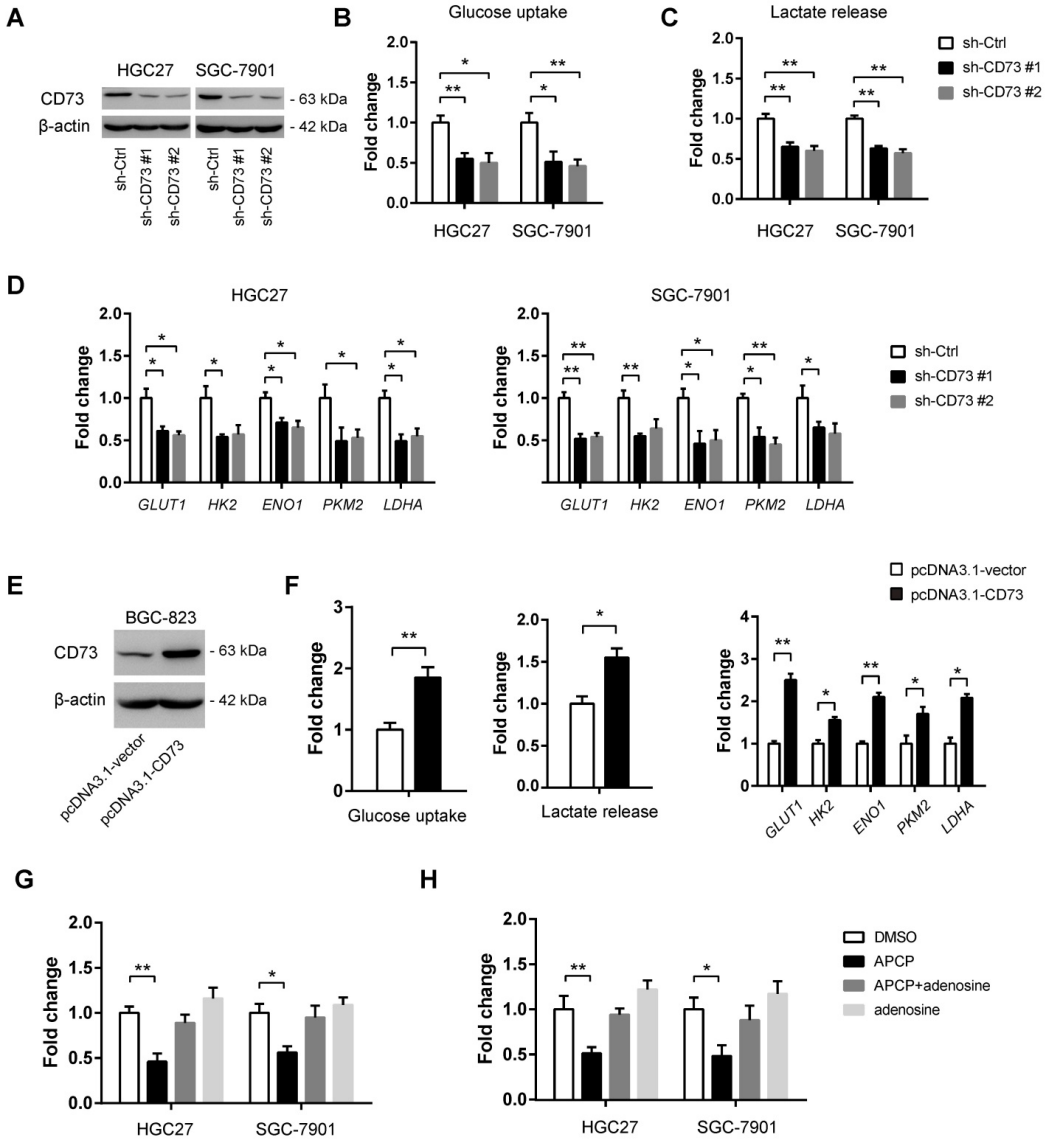
** CD73 regulates the Warburg effect in gastric cancer.** (**A**) The knockdown efficiency of two independent shRNAs against CD73 in HGC-27 and SGC-7901 cells was analyzed by western blotting analysis. (**B-D**) Effect of CD73 knockdown on the glucose uptake (B, n = 3), lactate release (C, n = 3), and expression of glycolytic genes (D, n = 3) in HGC-27 and SGC-7901 cells. (**E**) The overexpression efficiency of CD73 in BGC-823 cells was confirmed by western blotting analysis. (**F**) Effect of CD73 overexpression on the glucose uptake (n = 3), lactate release (n = 3), and expression of glycolytic genes (n = 3) in BGC-823 cells. (**G**) The effect of APCP (50 μM) and adenosine (10 μM) on the glucose uptake of HGC-27 and SGC-7901 cells. (**H**) The effect of APCP (50 μM) and adenosine (10 μM) on the lactate release of HGC-27 and SGC-7901 cells. *p < 0.05; **p < 0.01.

To demonstrate whether CD73 plays a role in gastric cancer glycolysis, *in vitro* loss-of-function studies were first carried out. Two shRNAs against CD73 led to a > 70% reduction of CD73 protein expression in both HGC-27 and SGC-7901 cells (**Figure 3A**). Consistent with the knockdown efficiency, reduction in CD73 expression led to a significant decrease in glucose uptake (**Figure 3B**), lactate production (**Figure 3C**), and expression of glycolytic genes (**Figure 3D**) in HGC-27 and SGC-7901 cells. To further confirm the potential regulatory role of CD73 in gastric cancer glycolysis, we overexpressed CD73 in BGC-823 cells (**Figure 3E**). As expected, ectopic expression of CD73 enhanced the Warburg effect as evidenced by glucose uptake, lactate release, and expression of glycolytic genes (**Figure 3F**). To further investigate whether the promotive effect of CD73 on the cell glycolysis was dependent on its enzyme activity or was mainly the results of its non-enzymatic function, we used 50 μM APCP (the specific inhibitor of CD73 enzymatic activity) to block the CD73 enzyme activity. The results showed that APCP treatment inhibited cell glycolysis, which can be largely restored by the addition of adenosine (**Figure 3G and 3H**), indicating that CD73 regulated the Warburg effect via enzyme-dependent activity.

### The growth advantage induced by CD73 is glycolysis-dependent in gastric cancer

**Figure 4:**
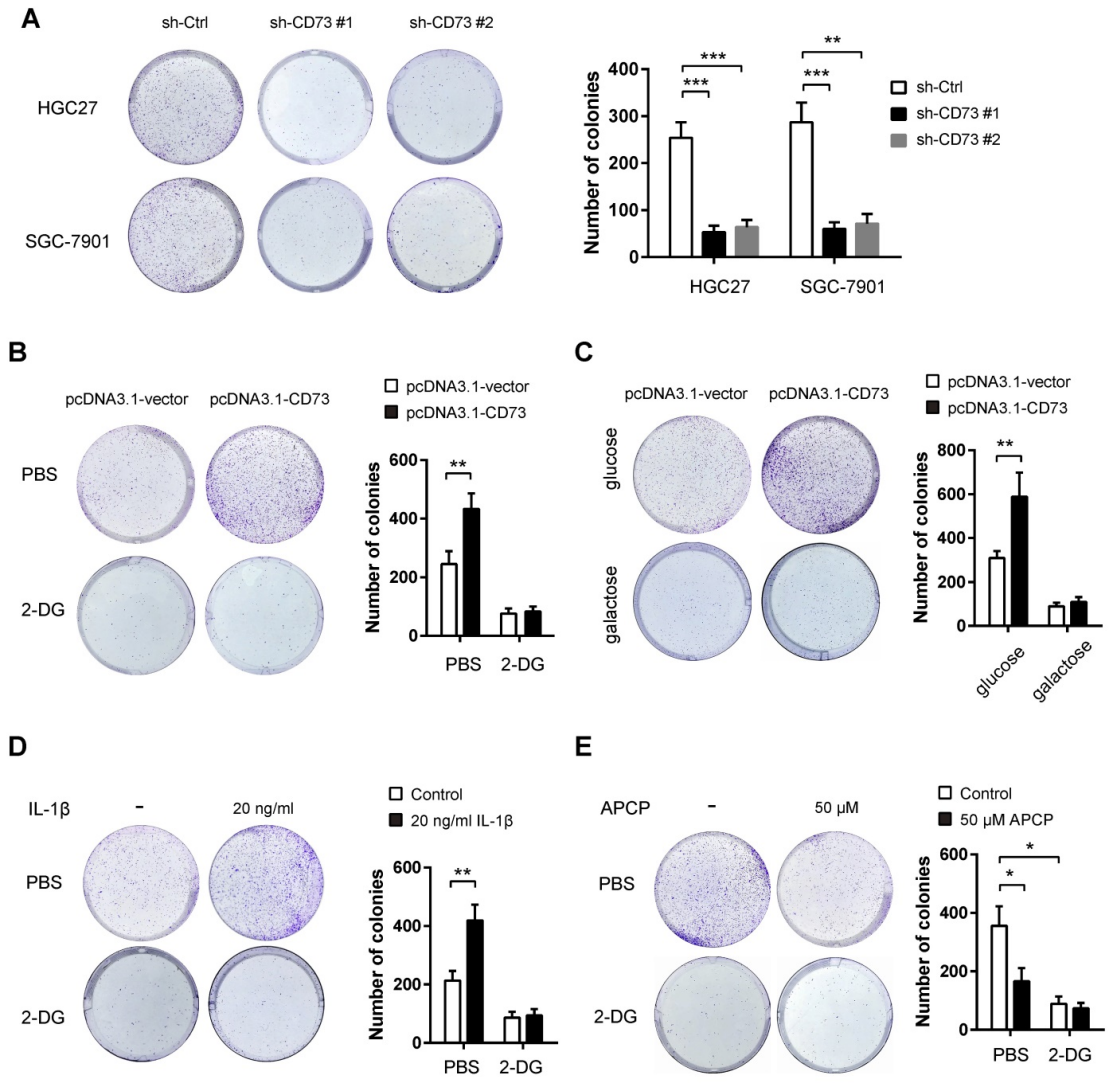
** The growth advantage induced by CD73 is glycolysis-dependent in gastric cancer.** (**A**) The effect of CD73 knockdown on *in vitro* proliferation of HGC-27 and SGC-7901 cells was analyzed by colony formation assay (n = 3). (**B**) The effect of CD73 overexpression on the proliferation of BGC-823 cells in the presence or absence of 50 mM 2-DG was analyzed by plate colony formation assay. (**C**) BGC-823 cells were cultured in media containing 25 mM galactose but no glucose and plate colony formation experiment was performed. (**D**) The effect of 2-DG on the proliferation of HGC27 cells in the presence or absence of 50μM APCP was analyzed by plate colony formation assay. **p < 0.01; ***p < 0.001.

Next, we studied the potential oncogenic roles of CD73 in gastric cancer. By plate colony formation assay, we found that CD73 knockdown contributed to decreased proliferative capabilities in HGC-27 and SGC-7901 cells (**Figure 4A**). Because that CD73 can promote the glycolytic ability of gastric cancer cells, we next tested whether CD73 affects tumor growth via modulation of tumor glycolysis. To address this hypothesis, we evaluated the effect of CD73 overexpression on gastric cancer cell proliferation. CD73 overexpression led to a significant increase in cell proliferation of BGC-823 cells, which can be largely abrogated by the addition of the known glycolysis inhibitor 2-DG (**Figure 4B**).Next, we grew BGC-823 cells in the culture medium containing galactose instead of glucose. The rate at which galactose enters glycolysis is much lower than that of glucose. Notably, galactose largely compromised CD73-mediated upregulation of cell proliferation (**Figure 4C**). Moreover, HGC27 cells were pretreated with glycolysis inhibitor 2-DG, followed by combined treatment with 50 μM APCP. As shown in the **Figure 4D**, 2-DG and APCP can suppress HGC27 cell proliferation, respectively. However, the tumor-suppressive effect was not further enhanced by combined treatment. Collectively, these data above suggest that CD73 couples cell glycolysis to tumor cell proliferation in gastric cancer.

### CD73 knockdown suppresses tumor growth *in vivo*

**Figure 5:**
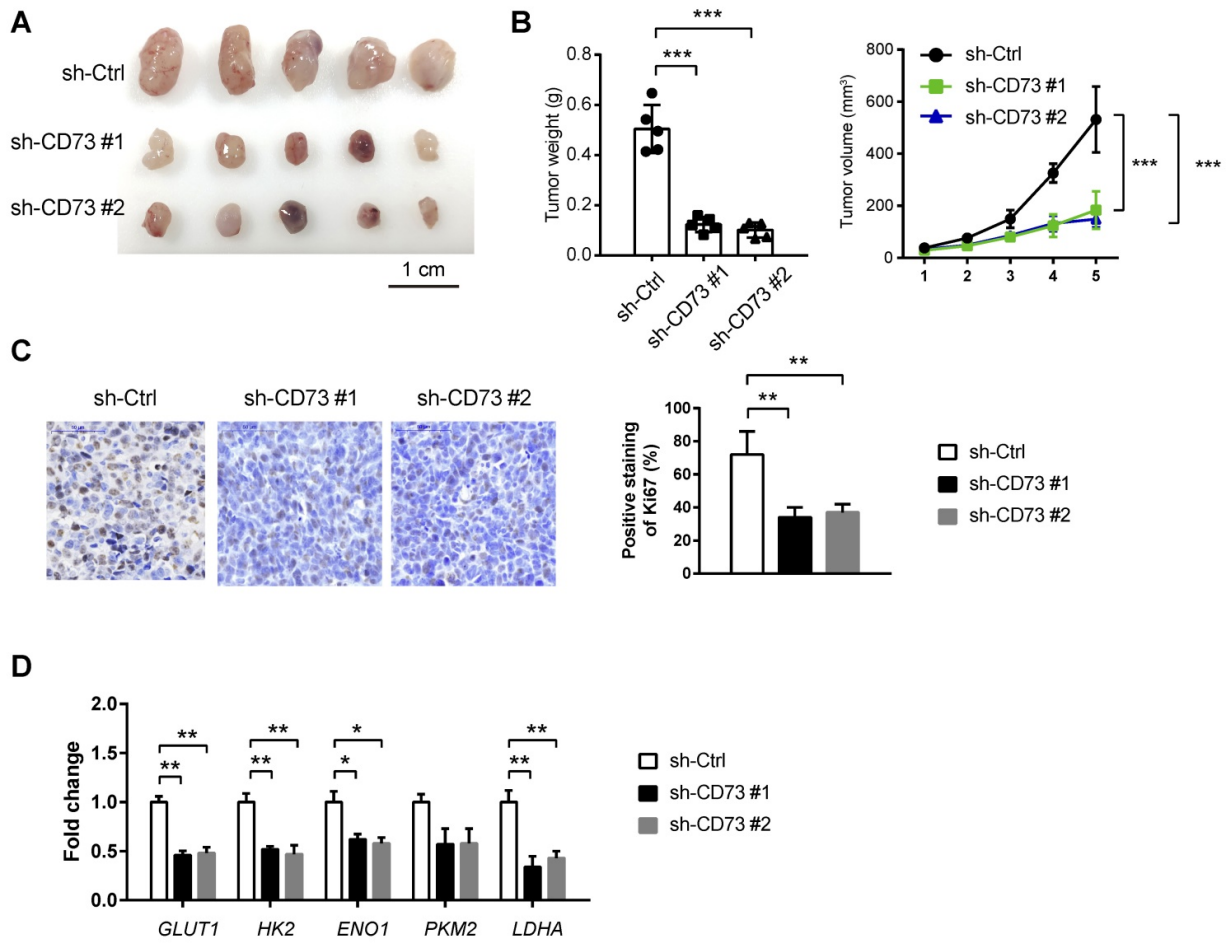
** CD73 knockdown suppresses tumor growth in vivo.** (**A**) A subcatenous xenograft model showed the effect of CD73 knockdown on the *in vivo* tumor growth of HGC-27 cells (n=5). (**B**) Measurement of tumor weight and tumor volume in sh-Ctrl, sh-CD73 #1 and sh-CD73 #2 groups. (**C**) IHC analysis of Ki67 in sh-Ctrl, sh-CD73 #1 and sh-CD73 #2 tumor tissues. (**D**) Real-time qPCR analysis of glycolytic genes (GLUT1, HK2, ENO1, PKM2, and LDHA) in sh-Ctrl, sh-CD73 #1 and sh-CD73 #2 tumor tissues; GLUT1, glucose transporter type 1; HK2, hexokinase 2; ENO1, enolase 1; PKM2, pyruvate kinase M2; LDHA, lactate dehydrogenase A. *p < 0.05; **p < 0.01; ***p < 0.001.

To test the *in vivo* effect of CD73 knockdown on tumor growth, a subcutaneous xenograft model was generated. As a result, stably knockdown of CD73 significantly retarded tumor burden as evidenced by tumor weight, tumor volume, and the proliferation index Ki67 (**Figure 5A-C**). By real-time qPCR analysis, we found that CD73 knockdown can lead to significant down-regulation of glycolytic genes (*GLUT1*, *HK2*, *ENO1*, and *LDHA*) in tumor tissues from the subcutaneous xenograft model (Fig. 5D).

## Discussion

CD73 plays diverse role in human cancers. In this study, we investigated (i) the glycolysis-associated genes in gastric cancer, (ii) the effect of CD73 on tumorigenic potential and the Warburg effect, (iii) whether increased growth advantage induced by CD73 is glycolysis-dependent, and (iv) whether CD73-mediated glycolysis is dependent on its enzyme activity. Our results suggest that CD73 is profoundly implicated in the Warburg effect and can be exploited as a potential target for gastric cancer therapy.

Aberrant expression of CD73 has been reported in many cancers, such as lung cancer, colorectal cancer, breast cancer, prostate cancer, ovarian cancer, pancreatic cancer, and gastric cancer 21-26. However, little is known about the reason for CD73 dysregulation. Previously, CD73 is identified as a target of miR-30a-5p and plays an important role in the pathogenesis of non-small cell lung cancer 27. Moreover, accumulated evidence suggests the link between CD73 and hypoxia. For instance, high CD73 expression in lung cancer cells is directly linked with hypoxia-inducible factor HIF1α expression by cancer cells 28; in fulminant acute liver failure 29, CD73 expression can be increased by HIF1α; in gastric cancer, CD73 expression correlates closely with HIF-1α expression 18. Consistent with these observations, we provided proof of principle data of hypoxia-dependent expression of CD73 in gastric cancer. Moreover, our findings further broaden the roles of CD73 in gastric cancer and suggest a glycolysis-dependent effect of CD73. Targeting CD73 by genetic inhibition or monoclonal antibodies might be a preferred way. In gastric cancer, inhibition of CD73 in primary tumors could dramatically abolish the effects of CD73 and eventually suppress experimental metastasis in both peritoneal seeding and hematogenous metastasis model 16. Moreover, recent studies have revealed an enhanced antitumor effect of anti-PD-1 or anti-CTLA-4 mAb in a mouse model when combined with anti-CD73 mAb 30, 31. Therefore, our and other studies support the possibility of targeting CD73 from diverse perspectives.

Emerging evidence suggests that CD73 is involved in the tumor growth and metastasis of many types of cancer, dependent on its enzyme activity in the production of adenosine in the tumor microenvironment. It has been reported that increased concentration of extracellular adenosine, which was always elevated by oxidative stress, correlated with immune and inflammation response via activation of immunosuppressive Tregs or inhibition of antitumor immune cells. CD73-mediated adenosine generation has been recognized as important purinergic signaling during cancer development and progression, which can suppress the immune response and assist tumor immune evasion in vivo via multiple signaling pathways. For example, the extracellular metabolism of ATP into adenosine through the CD38-NPP1-CD73 axis, the CD39-CD73 axis, and the NDPK/NME/NM23 axis is well-documented target system for tumor treatment 32. CD73 could be a critical regulator that promotes tumor progression in an immune-independent manner, such as tumor stemness, epithelial-to-mesenchymal transition (EMT), and therapy resistance 32, 33. Our data supported the concept that CD73-induced generation of adenosine promotes the Warburg effect and tumor growth in gastric cancer. Indeed, pharmacological inhibition of CD73 with APCP reduced glucose uptake and lactate release of gastric cancer cells. When tumor glycolysis was inhibited by 2-DG, APCP failed to induce an additional tumor-suppressive effect on gastric cancer cells. Acquisition of sufficient cellular buildings via the glycolysis pathway had been regarded as prerequisites for tumor growth. Here we provide a novel view of the growth-promoting roles of CD73 in gastric cancer. However, the detailed mechanism by which CD73 regulates the expression of glycolytic genes warrants further investigation.

## Conclusions

In conclusion, we report here that the hypoxic tumor microenvironment may induce CD73 expression in gastric cancer. CD73 facilitates the Warburg effect via its enzyme activity to produce adenosine. Given the important roles of the Warburg effect in facilitating tumor growth, these findings further support the pursuit of CD73 as a target for cancer therapy in gastric cancer.

## Abbreviations

TCGA: The Cancer Genome Atlas
GTEx: Genotype-Tissue Expression
2-DG: 2-deoxy-D-glucose
shRNAs: short hairpin RNAs
CM: conditioned medium
STAD: stomach adenocarcinoma
HIF1A: hypoxia-inducible factor-1alpha
GLUT1: glucose transporter type 1
HK2: hexokinase 2
ENO1: enolase 1
PKM2: pyruvate kinase M2
LDHA: lactate dehydrogenase A
